# Doors to the Homes: Signal Potential of Red Coloration of Claws in Social Hermit Crabs

**DOI:** 10.1093/iob/obad018

**Published:** 2023-05-22

**Authors:** C T M Doherty, M E Laidre

**Affiliations:** Department of Biological Sciences, Graduate Program in Ecology, Evolution, Environment, and Society, Dartmouth College, Hanover, NH 03755, USA; School of Health Sciences, Ulster University, Belfast BT15 1ED, UK; Department of Biological Sciences, Graduate Program in Ecology, Evolution, Environment, and Society, Dartmouth College, Hanover, NH 03755, USA

## Abstract

Red coloration on a signaler's body may be an informative signal in many animals. For species that inhabit architecture (e.g., burrows, nests, or other structures), certain parts of the body are more exposed than others, potentially serving as superior platforms for signaling via coloration. Yet whether animals differentially advertise red coloration on body parts that are more versus less exposed from their architecture remains to be tested. Here, we systematically quantified red coloration in social hermit crabs (*Coenobita compressus*). These crabs inhabit architecturally remodeled shells and have claws that visibly block the shell entrance, like doors to their homes. We hypothesized that red coloration of claws may be a signal of resource-holding potential (RHP). Consistent with this RHP signaling hypothesis, we found that within the same individuals’ bodies, exposed claws showed significantly greater red coloration than unexposed carapaces. Furthermore, larger body size predicted greater red coloration of claws. Competing hypotheses (e.g., interspecific signaling, camouflage, and UV protection), while not explicitly tested, nevertheless appear unlikely based on natural history. Red claw coloration may therefore function as a signal to conspecifics, and experiments are now needed to test recipient responses. Broadly, relative to surrounding architecture, exposed body surfaces offer rich potential as signaling platforms for coloration.

## Introduction

Animal signals are acts or structures produced by signalers that convey information (Bradbury and Vehrencamp 2011). For signals to convey information about the signaler, the signal must correlate with an underlying trait of the signaler that elicits a response in the receiver, which on average results in increased fitness of both the signaler and receiver (Searcy and Nowicki 2005; Laidre and Johnstone 2013). Often, substantial variation of traits exists within a population, but only signals have been specifically selected over evolutionary time to convey information about traits. Even when signals convey information imperfectly, they can still be useful, especially when combined with other information sources. Indeed, many multimodal signals (i.e., communication that incorporates signal components from two or more sensory modalities; Stevens 2013) work in such a way. While signals from many modalities can be informative, for species with vision, color may be of great significance (Kelber and Osorio 2010).

Red coloration, in particular, may be a potentially relevant signal in multiple contexts. For example, in fighting contexts, red has been found to be an indicator of signalers’ resource-holding potential (RHP) [e.g., in red-shouldered widowbirds, *Euplectes axillaris*, red coloration of epaulets signals competitive ability (Pryke and Andersson 2003a, b)]. RHP is an individual's ability to win and maintain possession of a contested resource (Parker 1974). Measures of RHP can include size (e.g., Reddon et al. 2011), weaponry, skill (e.g., Briffa and Fortescue 2017), strength and stamina (Jennings et al. 2005), and anything else that may enable an animal to prevail in a contest (Maynard Smith 1982). Assessment of RHP by competitors can comprise pure self-assessment, cumulative assessment, or mutual assessment [for more details on assessment models, see Parker (1974), Arnott and Elwood (2009), and Elwood and Arnott (2012)]. Yet, regardless of the assessment type, signals of RHP, such as red coloration (Pryke and Andersson 2003a, b), can evolve if the information conveyed can reduce the cost of fights for both signalers and receivers.

In addition to signals that convey RHP, examples of red color signals also exist in sexual selection as indicators of mate attractiveness [e.g., in zebra finches, red coloration of beaks determines sexual attractiveness (Blount et al. 2003)]. Other correlates of red coloration are immune function and antioxidant activity (Bendich and Olson 1989; Hill 1999; Alonso-Alvarez et al. 2004), as well as UV protection (Saikawa et al. 2004; Caramujo et al. 2012). One reason red coloration is thought to be a potentially useful signal across many taxa is that certain pigments that generate red coloration (e.g., carotenoids) cannot be synthesized *de novo* by animals (Goodwin 1992) and must instead be obtained from food in the external environment (Olson and Owens 1998; Hill 2000; McGraw and Ardia 2003). Therefore, individuals with a differential ability to acquire these pigments (e.g., through more efficient discovery or a greater capacity to dominate at resource patches) will display more red. Effectively conveying this information may depend on the precise placement of red coloration on the signaler's body.

It is generally physically impossible for organisms to display all areas of their bodies at once. Thus, while some species exhibit full-body red coloration (e.g., male Northern cardinals), most animals with red coloration only have specific areas of their bodies on which red is more concentrated (e.g., the faces of mandrills, the epaulets of red-winged blackbirds, and the dewlaps of Anolis lizards). Interestingly, organisms that inhabit architecture (e.g., burrows, nests, or other built structures) only have certain parts of their bodies that are regularly exposed (Laidre 2018; 2021a), typically at the architecture's entrance [e.g., mantis shrimp (Green and Patek 2018), phragmotic-headed ants (Wheeler 1927), woodpeckers (Paclík et al. 2022), and pine martens (Zalewski 1997)]. Such partial exposure often occurs when owners are protecting their architectural structures from intruders, with the owner keeping the majority of its body inside the structure for protection (Laidre 2021a). Exposed body surfaces of owners, relative to their architecture, may make ideal platforms for signaling via coloration. Yet, whether animals differentially advertise red coloration on body parts that are more versus less exposed from their architecture remains to be tested.

**Fig. 1:**
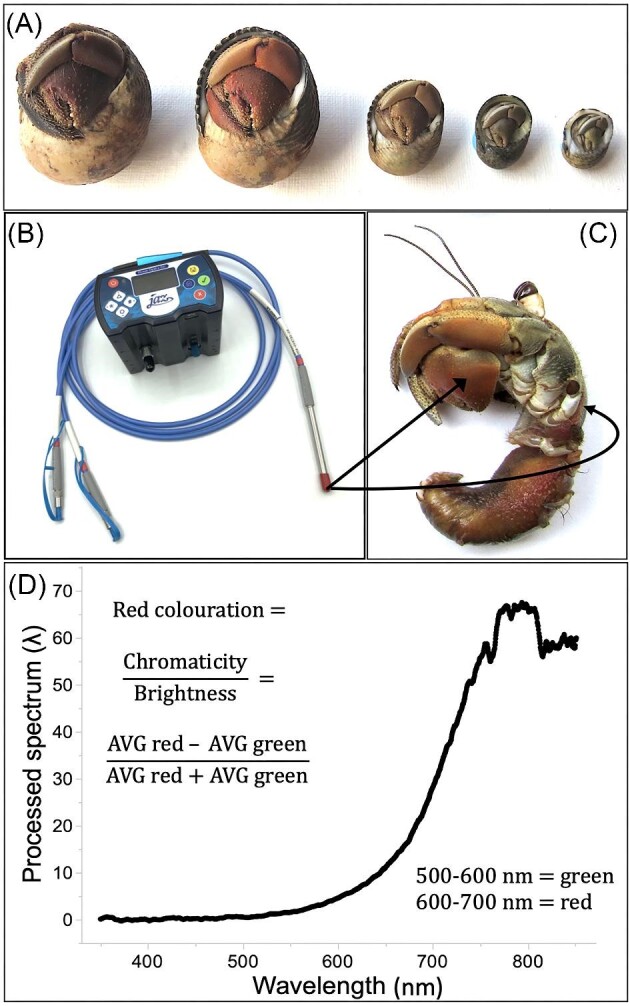
Measurements of body color, in relation to shell architecture, within the social hermit crab (*Coenobita compressus*). (**A**) Photograph of color variation across individuals (arrayed from largest to smallest from left to right). Note that only part of each individual's body, particularly its enlarged left claw, is exposed out of the shell architecture, being visible as a “door to the home.” (**B**) Reflectance was measured using a Jaz spectrometer. (**C**) Color measurements focused on two areas of crabs’ bodies: exposed claws (arrow on left) and unexposed carapaces (arrow on right). Individual pictured has been removed from its shell to show both areas. (**D**) Example of a reflectance curve for the claw of a male, including the formula used to calculate red coloration. (Photos for A and C: Mark Laidre; photo for B: Elliott Steele).

The organisms with perhaps the most intimate connection to the architecture they inhabit are hermit crabs (Laidre 2011). These animals’ bodies are mostly covered by transportable gastropod shells (Laidre 2012a), except for their anterior appendages, particularly claws, which represent a first line of defense of shells (Briffa et al. 1998; Arnott and Elwood 2010). Interestingly, for highly social, terrestrial hermit crabs (*Coenobita* spp.), the evolutionary transition to living on land (Burggren and McMahon 1988) was accompanied by dramatic changes in both sociality (Laidre 2012b, 2014, 2019a, 2021b) and claw morphology (Laidre 2021c). These social hermit crabs no longer produce threat displays with their claws (Doherty and Laidre 2020). Instead, their enlarged left claw now fits the shell entrance, essentially functioning as a “door” or “pseudo-operculum” (Grubb 1971; Abrams 1978; Doherty and Laidre 2020; Laidre 2021c), which is visible to conspecifics and blocks potential evictors (Laidre 2021b). Within one of these social hermit crab species (*Coenobita compressus*), the level of red coloration of claws varies greatly between individuals in the wild (Fig. 1A; Ball 1972). Yet no studies have systematically quantified coloration to test possible functional explanations. We hypothesized that red coloration on exposed body parts may be a potential signal, with the intensity of red providing information about RHP.

To test the hypothesis that red coloration serves as a signal of RHP, we systematically measured color variation between different parts of the body for individuals spanning a range of sizes and both sexes. If the RHP signaling hypothesis is true, then exposed areas should differentially advertise red coloration compared to unexposed areas of the same individuals’ bodies. Furthermore, if red coloration is a signal of RHP, then variation in red should correlate with body size, such that bigger individuals are redder in those exposed regions. Also, the sex that differentially benefits from attaining a larger size [which is males in *C. compressus* (Contreras-Garduño and Córdoba-Aguilar 2006; Contreras-Garduño et al. 2007); see also Supplementary Fig. S1] may be more likely to show a relationship between red coloration and body size. We therefore examined between-sex variation, assessing whether males showed a stronger relationship than females between red coloration and body size. We also evaluated variation (based on residuals in exposed surface area, i.e., claw area, relative to body size) to determine if individuals with greater exposed surface area, relative to body size, were more red. If variation in red coloration is unrelated to RHP, then the above predictions should not follow. Note that red coloration could convey information as either a signal, which has been specifically selected to convey that information, or as a cue. Cues, like signals, also convey information and are likewise correlated with conditions of interest to receivers, though cues have not been specifically selected to convey information (Bradbury and Vehrencamp 2011). Here we use signal as a shorthand, and we consider in the discussion further criteria needed to tease apart signal and cue. We also consider the extent to which any alternative hypotheses, adaptive or non-adaptive, may explain the observed patterns in coloration.

## Methods

### Sample collection and study site

*Coenobita compressus* specimens were collected in the wild from a long-term study population (Laidre 2010) along the beach-forest interface of the Osa Peninsula, Costa Rica (8°23′40′′ N, 83°20′10′′ W). Individuals of all sizes across the population were collected. Collection was entirely blind with respect to color and sex. The sole criteria for inclusion were that an individual must have all its appendages intact and not be moulting. Once the sample (*N* = 103 individuals) was collected, all individuals were then systematically measured. To test if body parts that are exposed out of the shell architecture could act as signaling platforms, a series of systematic morphological and color measurements (see below) were taken on each individual. All measurements were made by the first author (C.D.) during February and March 2018. After measurements were completed, all collected individuals were then returned to the wild.

### Morphological measurements

For each individual, we recorded sex [*N* = 47 females, *N* = 56 males; determined using the standard method as described in Laidre (2019b)], and made the following morphological measurements using electronic callipers to the nearest 0.01 mm: shield length (mm) (Laidre 2019b), claw length (mm) (Laidre 2021c), and claw width (mm) (Laidre 2021c). We also calculated exposed claw area (mm^2^) as claw length × claw width. In addition, we measured posterior carapace length to the nearest 0.01 mm and body weight to the nearest 0.01 g. However, because shield length is regarded as the most reliable metric of overall body size (Laidre 2019b) and also because shield length correlated strongly with both posterior carapace length and body weight (Supplementary Table S1), all our analyses of body size focus on shield length.

### Color measurements

We measured spectral reflectances (from 250 to 800 nm) using a solarization-resistant reflectance probe (QR400-7-SR reflection probe, Ocean Optics Inc., Dunedin, FL) coupled with a pulsed xenon source (Ocean Optics) and a JAZ spectrometer (JAZ-COMBO, Ocean Optics; Fig. 1B). Reflectance measurements were taken on two body parts for each individual (Fig. 1C): (1) the enlarged left claw (i.e., the body part that is most exposed and visible at the shell opening) and (2) the carapace (i.e., the body part that is concealed within the shell and only becomes visible after an individual is removed from its shell). Both body parts represent hardened areas of the same overall exoskeleton as opposed to the soft fleshy tissue of the abdomen. Note that we did not make any color comparisons with the smaller right claw or the walking legs since these body parts were too small to use spectrometer measures, and furthermore, they are neither fully concealed nor fully exposed, so they would not have provided informative comparisons. Only the enlarged left claw and the carapace, which were possible to measure, could provide a direct and informative test of the RHP signaling hypothesis.

Reflectance measurements were calibrated using a Spectralon plastic standard that reflects nearly 100% of the light at all wavelengths from 200 to 800 nm (WS-1-SL Diffuse Reflectance Standard, Ocean Optics), which allows the spectrometer to calculate processed spectrum values (i.e., reflectance corrected using the white reference; e.g., see Fig. 1D). To control for variation caused by the angles of illumination or measurement, a reflection probe holder (RPH-1) was used to ensure the end of the reflectance probe was always placed at the same distance from the measured surface and held at a consistent 45° angle to the surface. This arrangement, which uses an angle of collection that is not equal to the angle of incident light, measures diffuse reflectance (which is relatively angle-independent), and avoids detection of specularly reflected light (Johnsen 2005).

For each body part, we quantified red coloration using the following formula (Stevens et al. 2014; Fig. 1D):


\begin{eqnarray*} \frac{\left( {\rm average\ red - average\ green} \right)}{\left( {\rm average\ red + average\ green} \right)} \end{eqnarray*}

This formula calculates chromaticity (i.e., average red−average green) while controlling for brightness (i.e., average red + average green) and uses measurements in the 500–600 nm wavelength range to denote the green spectrum and measurements in the 600–700 nm wavelength range to denote the red spectrum.

### Final sample

Of the original sample (*N* = 103 collected individuals), a subset was excluded from the analyses of color for the following reasons: *N* = 1 individual had an abdominal abnormality, which was only detectable after it had been removed from its shell; *N* = 21 individuals were too small to accommodate the spectrometer to take color measurements; *N* = 9 individuals were large enough to take color measurements, but ultimately their spectrometer files could not be opened or were somehow corrupted. Hence, analyses of color were performed on a final sample of *N* = 72 individuals (*N* = 28 females and *N* = 44 males).

### Statistical analyses

To examine whether red coloration could function as a signal, we assessed the factors related to red coloration using a generalized linear mixed model (GLMM), with measures of red coloration being the response variable. We included a full factorial of body part (exposed or unexposed), sex (male or female), and body size (shield length) as fixed effects. We also included crab identity as a random effect in the model to account for multiple measurements per individual (i.e., one measurement on the exposed body part and one measurement on the unexposed body part of the same crab). All crabs in this study were collected in the same geographic vicinity, within an approximately 10–20 m stretch of beach, which crabs can readily traverse. Hence, geographic location was not considered a variable in the model. Finally, to test if coloration was related to the size of the exposed claw area relative to body size, we conducted a post-hoc linear regression of red coloration of claws against the residuals of exposed claw area relative to shield length. All analyses were performed in JMP® Pro 16.0.0.

## Results

**Fig. 2:**
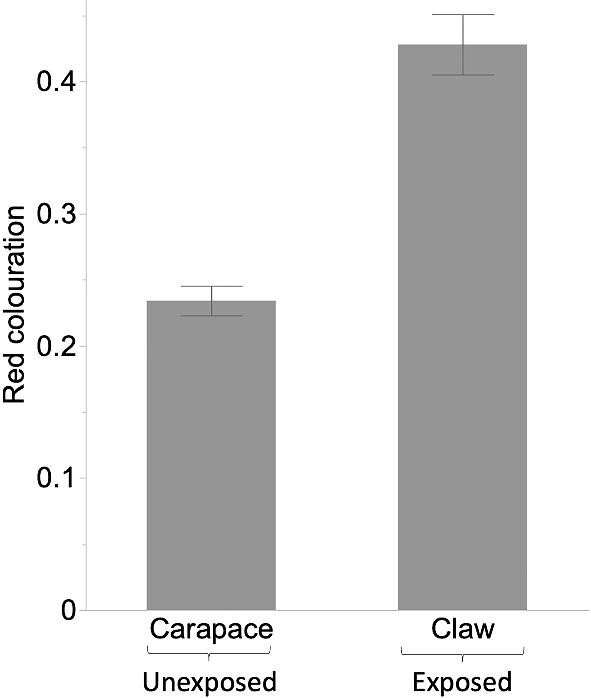
Red coloration (mean ± SE) of exposed claws versus unexposed carapaces for *N* = 72 individuals.

**Fig. 3:**
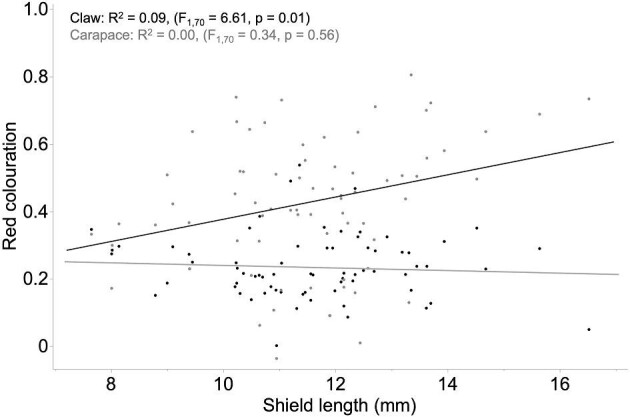
Interaction plot of red coloration of body parts against overall body size (shield length in mm) for *N* = 72 individuals. Exposed claws are shown with black dots and black trendline, and unexposed carapaces are shown with gray dots and gray trendline.

Consistent with the main prediction of the RHP signaling hypothesis, within-subject contrasts revealed a significant difference in red coloration between exposed versus unexposed body parts (i.e., claw versus carapace within the same individuals). In particular, claws showed significantly more intense red coloration than carapaces (GLMM: *F*_1,68_ = 55.98, *P* < 0.0001; Fig. 2), almost twice the intensity. Further aligning with the RHP signaling hypothesis, we found a marginally significant interaction between body part (exposed or unexposed) and body size (GLMM: *F*_1,68_ = 4.07, *P* = 0.0477). In particular, a positive relationship existed between body size and red coloration of claws (*r*^2^ = 0.09, *P* = 0.01; Fig. 3), but not for carapaces (*P* = 0.56; Fig. 3). There was no significant effect of sex on red coloration (GLMM: *F*_1,68_ = 1.08, *P* = 0.30).

**Fig. 4:**
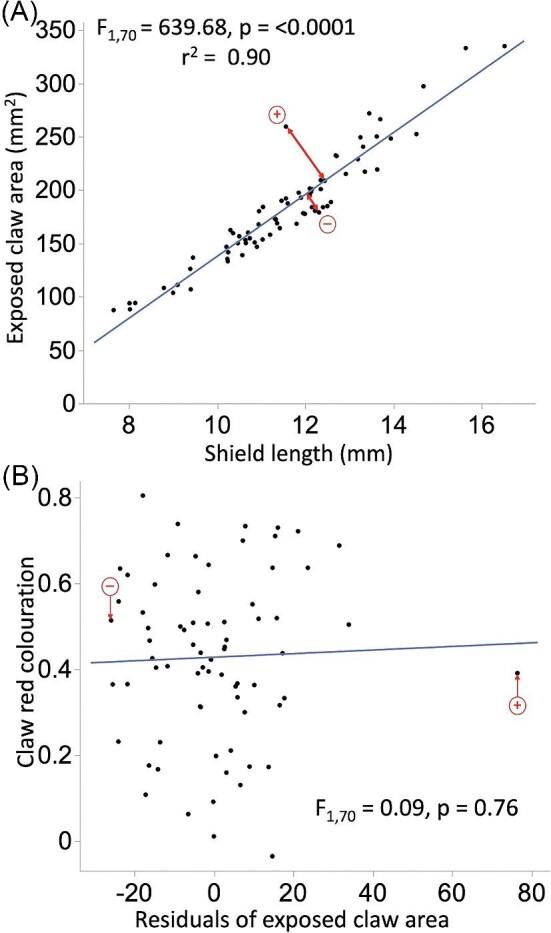
(**A**) Linear regression of exposed claw area (mm^2^) against overall body size (shield length in mm) for *N* = 72 individuals. Red arrows show residuals (most positive and most negative) for differences from the predicted line. (**B**) Linear regression of red coloration of exposed claws against residuals of exposed claw area. Related data points highlighted in (**A**) and (**B**) for reference.

Exposed claw area strongly correlated with body size (Fig. 4A; Supplementary Table S1). However, red coloration of claws did not correlate with variation in exposed claw area relative to body size (linear regression: *F*_1,70_ = 0.09, *P* = 0.76; Fig. 4B). Thus, individuals with proportionately more exposed surface area did not show greater red coloration.

## Discussion

Our systematic measurements of coloration and morphology among social hermit crabs revealed a stark difference in the level of red coloration between exposed and unexposed areas of the body, relative to architecture. In particular, exposed claws exhibited substantially more intense red coloration than unexposed carapaces, suggesting that red coloration could be a signal. Moreover, red coloration of claws increased with body size, suggesting that the level of red coloration in claws could be a signal of RHP. While the relationship between red coloration of claws and body size is relatively weak, red coloration could still provide valuable information for receivers by supplementing other sources of information about RHP [e.g., visual motion (Laidre 2013a), olfaction (Valdes and Laidre 2019), tactile feedback (Bates and Laidre 2018), and size assessment (Steele and Laidre 2023)]. Notably, one reason the relationship between color and body size may have been weak is because measurement of coloration was impossible for the smallest individuals sampled (20.4% of the original sample of *N* = 103 individuals; see Supplementary Table S1 and Fig. S2). Individuals of this small size, which were too small to accommodate the spectrometer, tend to exhibit little if any red coloration (Ball 1972; M.L. and C.D. personal observation). Thus, the relationship we found between red coloration of claws and body size might have been stronger if it had been possible to include the smallest individuals. The prediction of the RHP signaling hypothesis regarding sex was not supported in the current study, suggesting that even if attaining a larger size is differentially important for one sex (Supplementary Fig. S1), it may not be sufficient to favor differential color between sexes, perhaps due to competition for resources other than mates [e.g., at food patches (Laidre 2013a)]. A relationship between the residuals of exposed claw area and red coloration of claws was not found. Such a relationship may have been impossible given the extremely tight correlation between exposed claw area and body size (*r*^2^ > 0.9), which greatly restricted the range of residuals. Regardless, exposed areas could effectively advertise important information about RHP. Our results suggest that further tests of such possible signaling platforms on the body, in reference to architecture, are merited.

Further study of the pattern of red coloration we found will also be vital for testing potential alternative explanations that do not invoke intraspecific signaling. These alternative explanations (see Table 1), which cannot all be eliminated at present, include both adaptive and non-adaptive explanations. For example, one adaptive explanation is that red coloration of claws is used for interspecific signaling to predators. However, predation on land is relaxed for terrestrial hermit crabs (Vermeij 1993), and, furthermore, the architecturally remodeled shells used by our study species break at forces greater than their terrestrial predators are capable of producing (Laidre et al. 2012). Hence, explanations relating to interspecific signaling [e.g., aposematism (Pawlik 1993; Nishida 2002)], as well as camouflage against predators (Cuthill et al. 2006a, b, Stevens and Cuthill 2006; Stevens et al. 2014), are unlikely. Another adaptive explanation for the greater red coloration of claws is that red coloration is used for UV protection of exposed areas (Auerswald et al. 2008). However, the study species carries its claws ventrally (Ball 1972; Laidre 2012a) in the shade of both its body and shell while locomoting (Trinh and Laidre 2016), so the claws are the least exposed to the sun; furthermore, individuals move to forested areas (Laidre 2010) and beneath leaves (Steele and Laidre 2019) during the day. Hence, explanations relating to UV protection are also unlikely. Interspecific signaling, camouflage, and UV protection, while unlikely explanations for the difference in red coloration between exposed claws and covered carapaces, can still be formally tested in the future. Also, non-adaptive explanations remain possible. For example, direct environmental impacts and relative exposure to external elements (e.g., weather, sand abrasion, salinity, or sunlight) could perhaps cause the different coloration of claws compared to carapaces, especially if such impacts accumulate over a crab's lifetime. Or, theoretically, differences in red coloration between claws and carapaces might arise as an incidental by-product of some other trait that is directly selected for besides color [e.g., morphological structures, such as integument thickness (Davies et al. 2014)]. More studies are needed to determine the plausibility of alternative adaptive and non-adaptive explanations and the extent to which they can better explain patterns of red coloration compared to the RHP signaling hypothesis that was the focus of the present study.

**Table 1:** Competing hypotheses for the pattern of red coloration

	Explanation	Supported?
***Adaptive***		
Intraspecific signal	Selected to convey information to conspecifics (e.g., signaler's RHP)	**Yes**
Interspecific signal	Selected to convey information to heterospecifics (e.g., signaler's toxicity)	**Unlikely^a^**
Camouflage	Selected to help individual evade predators by blending with background	**Unlikely^a^**
UV protection	Selected to absorb sunlight at damaging wavelengths	**Unlikely^a^**
***Non-adaptive***		
Direct environmental impact of exposure	Due to greater exposure to external elements (e.g., weather, sand abrasion, salinity, or sunlight) outside of architecture	**Unclear**
By-product of selection for another trait	Selection on a trait other than red coloration incidentally gives rise to this color pattern	**Unclear**

a Years of natural history observations and accumulated knowledge on the study system suggest that these explanations are logically quite unlikely (see Discussion). Nevertheless, further explicit tests, especially experimental tests of these and other hypotheses, would be valuable.

In addition to testing alternative hypotheses, more detailed tests of the RHP signaling hypothesis would also be informative. If this hypothesis is true, then multiple finer-grained measures of RHP should correlate even more strongly with the level of red coloration in claws. For example, muscles both in the claw and the abdomen are undoubtedly critical for obtaining and retaining shells (Laidre 2021b), thereby allowing individuals to successfully rise and maintain their positions in the housing market (Laidre and Vermeij 2012). Future studies could therefore measure both claw pinching force and physical resistance to eviction, testing how well red coloration predicts individual's ability to evict others and withstand eviction. Furthermore, physiological studies could quantify the density of muscle fibres within both claws and abdomens, testing how well such internal musculature measures (Taylor 2000) correlate with red claw coloration. Ultimately, if further correlations exist between a variety of measures of RHP and red coloration, then this would bolster the RHP signaling hypothesis.

A key criterion for a signal, including a signal of RHP, is that recipients must be able to perceive and hence respond to the signal (Searcy and Nowicki 2005; Bradbury and Vehrencamp 2011; Laidre and Johnstone 2013). Interestingly, laboratory experiments in terrestrial hermit crabs have revealed that individuals can differentiate artificial red coloration from blue and green coloration (Ping et al. 2015). However, the response to natural color variation in claws has yet to be tested. Future experiments could exploit such natural variation by using postured or withdrawn models of dead crabs (Doherty and Laidre 2020), thereby testing recipient responses in the wild, particularly when other factors (e.g., claw size and shell quality) are held constant. Experiments might also be able to manipulate claw color itself, thereby directly testing whether recipients’ attempts at evicting a target change in response to altered color. If the level of red coloration of claws is indeed an effective signal, then recipients should make fewer attempts at evicting the signaler and give up earlier when the signaler's claw coloration is more red. Beyond signaling RHP, it is also possible that red claw coloration could simultaneously be used in sexual signaling (Contreras-Garduño and Córdoba-Aguilar 2006; Contreras-Garduño et al. 2007), in which case tests of female responses to males would be informative. Recent discoveries in birds revealing that color perception can be categorical, with discrete thresholds, rather than operating along a continuum (Caves et al. 2018) raise many further questions about the use and effectiveness of red coloration as a signal across taxa. In theory, multiple strategies could also be at play, with some individuals investing heavily in red coloration while others invest little.

To fully elucidate the function of red coloration, it may also be critical to gain a deeper understanding of mechanism. Mechanistically, red coloration is often generated using pigments obtained from food (Goodwin 1992), most notably carotenoids, such as astaxanthins, in many crustaceans generally (Carlisle and Knowles 1959; see Table 2 in Ahmadkelayeh and Hawboldt 2020), with astaxanthins also having been shown to impact color in hermit crabs specifically (see Table 1 in Wade et al. 2017). Presently, it remains unknown which exact compounds in the diets of wild terrestrial hermit crabs contribute to their red coloration. Yet, interestingly, *C. compressus*, in particular, is a highly opportunistic omnivore with one of the most diverse diets of any crustacean (Laidre 2013b). If specific dietary components required to achieve red coloration are hard to obtain in this species’ environment, then variation in individuals’ ability to find and gain access to such foods could provide a mechanistic basis for the RHP signaling hypothesis for red coloration. Determining what, if any, components of diet contribute to the accrual and retention of red coloration in this species will require experiments with controlled feeding regimens. Future experiments can also separately consider crabs’ ages, which need not be equivalent to their body sizes, given that hermit crabs metabolically control their growth (e.g., Alcaraz and García-Cabello 2017; Alcaraz et al. 2020), growing only when they acquire larger shells. By simultaneously monitoring individuals’ coloration longitudinally, both within and across moults, it might be possible to reveal how individuals allocate color differentially to certain body parts. Interestingly, six hermit crab species, when grown in the lab on the same feeding regimes, all exhibited different coloration, with differences that persisted across ontogeny (Hamasaki et al. 2017). Such feeding experiments could be supplemented with nutritional analyses that isolate the exact chemical composition of a range of natural foods in the wild. Ultimately, understanding red coloration at the proximate level, including what compounds are involved, how limited those compounds are in nature, and how individuals incorporate these compounds, will further inform our understanding of potential functions of red coloration.

To summarize, here we tested the hypothesis that red coloration in claws serves as a signal of RHP. Several key predictions of this RHP signaling hypothesis have been supported by our systematic measurements of color and morphology in social hermit crabs. A subset of competing hypotheses (Table 1) appear unlikely based on natural history. Future studies can further test the RHP signaling hypothesis, as well as other hypotheses, with behavioral experiments on the responses of recipients to color variation being key. We suggest that studies of other animals, particularly those inhabiting architectural structures, consider exposed body parts, relative to architecture, as potential signaling platforms.

## Supplementary Material

obad018_Supplemental_Files

## Data Availability

All data available as Electronic Supplementary Material.
