# Supplementary material for: Doors to the Homes: Signal Potential of Red Coloration of Claws in Social Hermit Crabs
Source: Integr Org Biol. 2023 May 22;5(1):obad018. doi: 10.1093/iob/obad018 (PMC10263385; doi:10.1093/iob/obad018)
Supplement: obad018_Supplemental_Files [file obad018_supplemental_files.zip › IOB_Figure S1_24.04.23.docx]

**
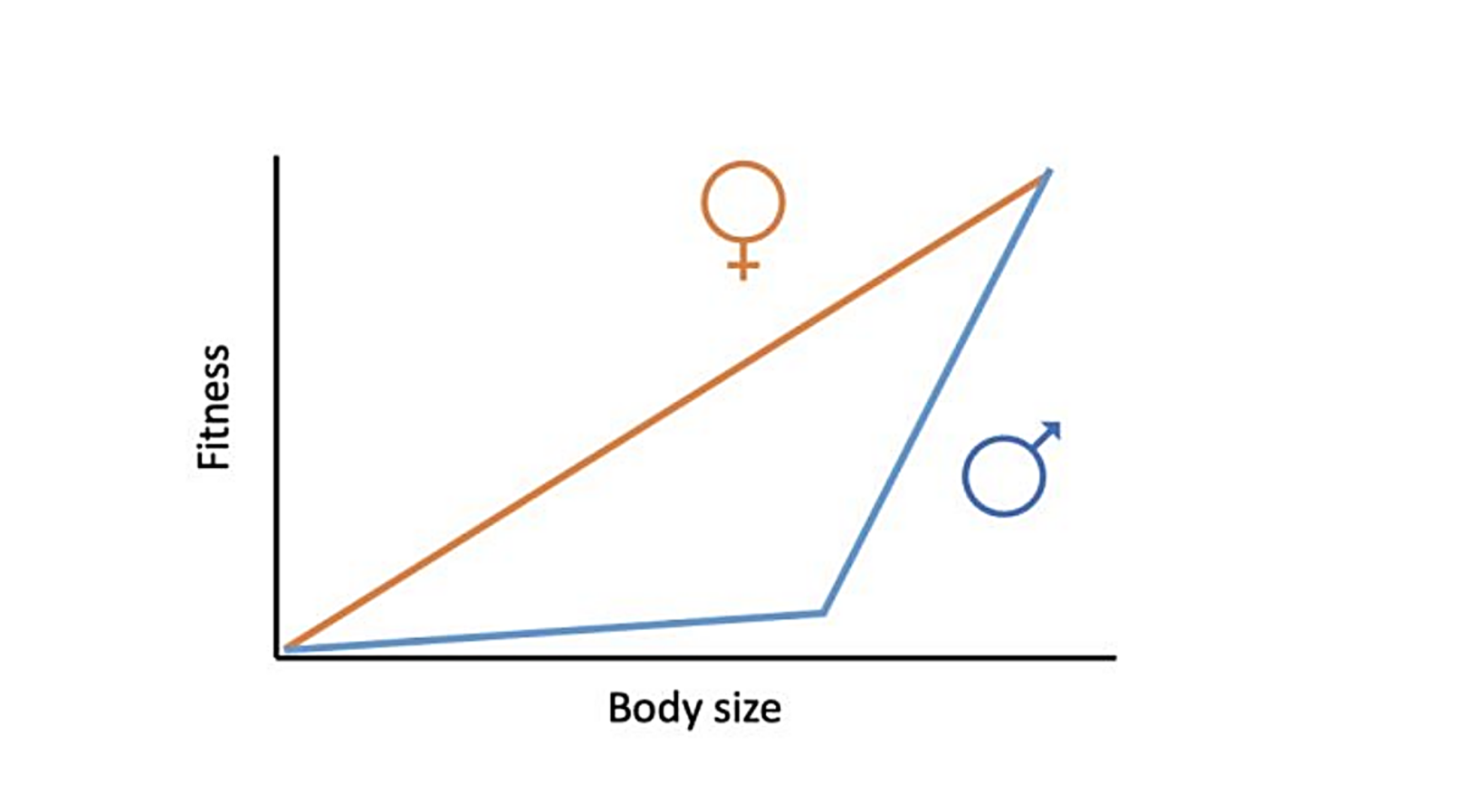
**

**Figure S1.** Conceptual figure illustrating the likely relationship between fitness and body size for each sex in hermit crabs (see [45, 46]). Female fitness increases linearly with body size, given that bigger females can carry more eggs inside their bigger shells. In contrast, males can only outcompete other males for mating opportunities once they reach a large enough threshold body size.
