# Supplementary material for: Doors to the Homes: Signal Potential of Red Coloration of Claws in Social Hermit Crabs
Source: Integr Org Biol. 2023 May 22;5(1):obad018. doi: 10.1093/iob/obad018 (PMC10263385; doi:10.1093/iob/obad018)
Supplement: obad018_Supplemental_Files [file obad018_supplemental_files.zip › Doors to the homes - Supplementary Table 1.docx]

**Table S1**. Correlation of each measure with overall body size (shield length in mm). Sub-categorized by individuals in the original sample and the final sample. Only individuals in the final sample were included in the colour analyses.

|  | **Sample size** | **p** | **r^2^** |  |  |  |  |
| --- | --- | --- | --- | --- | --- | --- | --- |
| **Original sample** |  |  |  |  |  |  |  |
| Weight | 103 | <0.0001 | 0.86 |  |  |  |  |
| Posterior carapace length | 103 | <0.0001 | 0.97 |  |  |  |  |
| Exposed claw area | 103 | <0.0001 | 0.96 |  |  |  |  |
| **Final sample (included in colour analyses)** | | | |  |  |  |  |
| Both sexes |  |  | | |  |  | |
| Weight | 72 | <0.0001 | 0.91 |  |  |  |  |
| Posterior carapace length | 72 | <0.0001 | 0.90 |  |  |  |  |
| Exposed claw area | 72 | <0.0001 | 0.90 |  |  |  |  |
|  |  |  |  |  |  |  |  |
| Males |  |  |  |  |  |  |  |
| Weight | 44 | <0.0001 | 0.93 |  |  |  |  |
| Posterior carapace length | 44 | <0.0001 | 0.90 |  |  |  |  |
| Exposed claw area | 44 | <0.0001 | 0.91 |  |  |  |  |
|  |  |  |  |  |  |  |  |
| Females |  |  |  |  |  |  |  |
| Weight | 28 | <0.0001 | 0.89 |  |  |  |  |
| Posterior carapace length | 28 | <0.0001 | 0.90 |  |  |  |  |
| Exposed claw area | 28 | <0.0001 | 0.90 |  |  |  |  |
|  |  |  |  | | | |  |
