# Supplementary material for: Doors to the Homes: Signal Potential of Red Coloration of Claws in Social Hermit Crabs
Source: Integr Org Biol. 2023 May 22;5(1):obad018. doi: 10.1093/iob/obad018 (PMC10263385; doi:10.1093/iob/obad018)
Supplement: obad018_Supplemental_Files [file obad018_supplemental_files.zip › Doors_to_the_homes_-_Supplementary_Figure_2.docx]

**
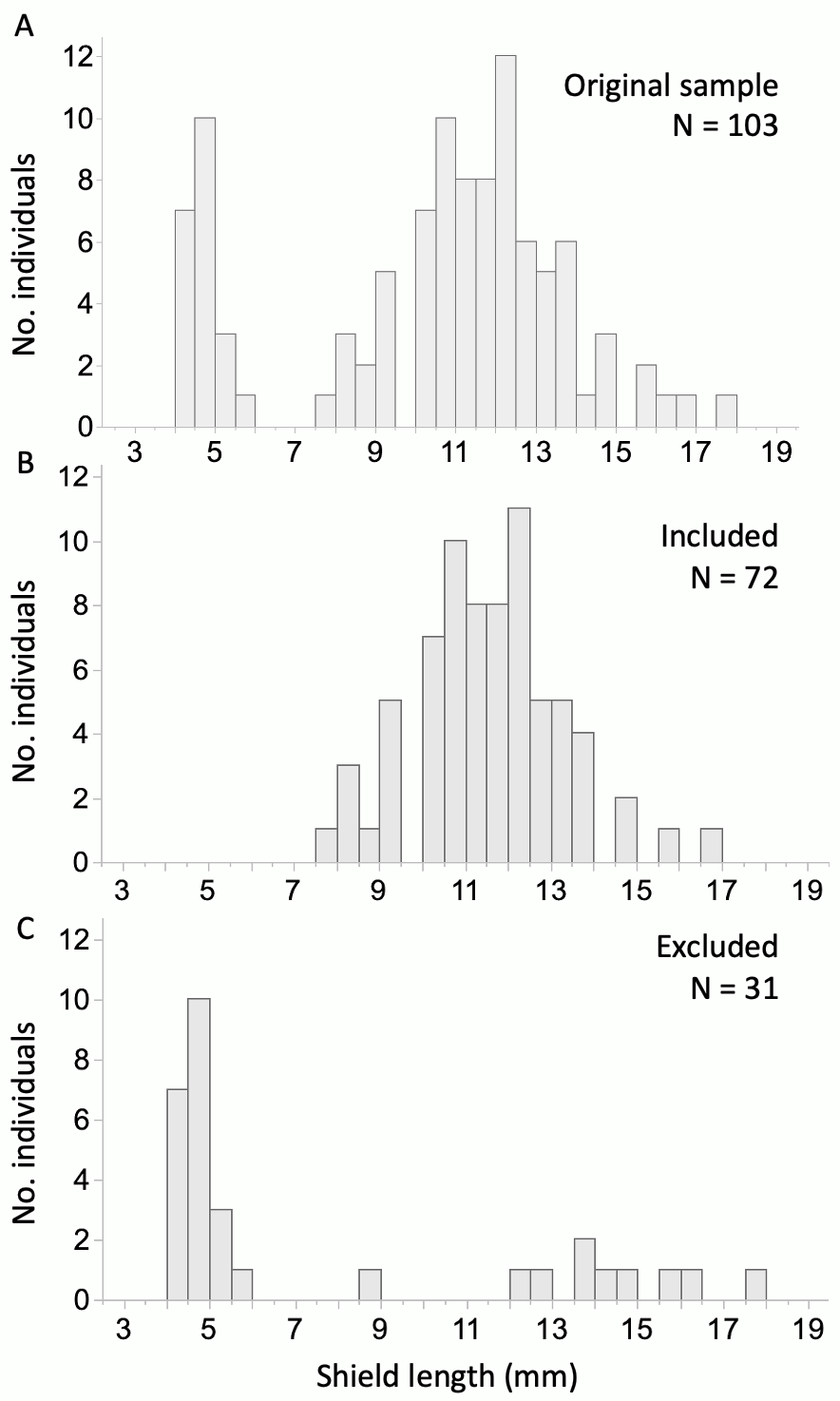
**

**Figure S2.** Distribution of overall body size (shield length in mm) for (A) all individuals in the original sample, (B) individuals in the final sample included in the colour analyses, and (C) individuals excluded from the colour analyses (see Methods for explanation).
